# Brief lifestyle interventions for prediabetes in primary care: a service evaluation

**DOI:** 10.1186/s12875-022-01658-2

**Published:** 2022-03-14

**Authors:** Rhys Thatcher, Nicholas Gregory, Wai Yee Cheung, Gareth J. Dunseath, Sharon N. Parsons, Mark Goodwin, Stephen D. Luzio

**Affiliations:** 1 Institute of Biological Environmental and Rural Sciences, Aberystwyth University, Aberystwyth, UK; 2 Public Health Wales, Tyndall Street, Cardiff, UK; 3 Diabetes Research Group, Swansea University, Swansea, UK; 4 AFAN Valley Practice, Glyncorrwg, Port Talbot, UK

**Keywords:** General Practice, Lifestyle, Prediabetic State, Prevention of Diabetes

## Abstract

**Background:**

The increasing number of cases of prediabetes in the UK is concerning, particularly in Wales where there is no standard programme of support. The aim of the current service evaluation was to examine the effectiveness of brief lifestyle interventions on glucose tolerance in people at risk of developing type 2 diabetes.

**Methods:**

In this pragmatic service evaluation clinical data on people deemed at risk of developing type 2 diabetes were evaluated from two GP clusters. Patients (*n* = 1207) received a single 15 to 30-min, face-to-face, consultation with a health care practitioner. Interventions were assessed by changes in HbA1c and distribution across the HbA1c ranges 12 months following intervention. Statistical significance of reversion to normoglycaemia and development of diabetes were assessed through comparison with expected rates without intervention.

**Results:**

Between baseline and 12-month follow-up HbA1c fell from 43.85 ± 1.57 mmol/mol (6.16 ± 0.14%) to 41.63 ± 3.84 mmol/mol (5.96 ± 0.35%), a decrease of 2.22 mmol/mol (0.20%) (95% CI 2.01 (0.18%), 2.42 (0.22%); *p* < 0.0001). The proportion of people with normal glucose tolerance at 12 months (0.50 95%CI 0.47, 0.52) was significantly larger than the lower (0.06 (*p* < 0.0001) and the upper (0.19 (*p* < 0.0001)) estimates based on no intervention.

**Conclusion:**

Results indicate significant improvement in glucose tolerance across GP clusters. The brief intervention has the potential to offer a robust and effective option to support people at risk of developing type 2 diabetes. Further research in the form of a randomised trial is needed to confirm this and identify those likely to benefit most from this intervention.

## Background

Under the Quality and Outcomes Framework (QOF) general practices register the number of people aged 17 years and over with diabetes. In 2020, Wales had the highest prevalence of diabetes in the UK with 209,015 people diagnosed, 8.0% of the population [1]. It is also estimated that a further 580,000 are at risk of developing type 2 diabetes and if the current increase in prevalence continues, 311,00 people in Wales will have diabetes by 2030 [1]. The burden of diabetes on the health care system is considerable with one in six of all people in hospital having diabetes [2].

Prediabetes is a metabolic condition which can develop into type 2 diabetes. It is characterised by the presence of blood glucose levels that are higher than normal but not high enough to be classed as diabetes. The NICE criteria for people at high risk of developing Type 2 diabetes are a HbA1c of 42–47 mmol/mol (6.0–6.4%) or fasting plasma glucose 5.5–6.9 mmol/L [3]. Currently, there is no standard programme of support for people with prediabetes in Wales.

The increasing number of new cases of prediabetes is concerning [4]. To help tackle this problem in England, Public Health England (PHE), NHS England and Diabetes UK have implemented the NHS Diabetes Prevention Programme (DPP) [5]. The NHS DPP consists of lifestyle management programmes and is aimed at those at risk of diabetes as defined by the NICE criteria [3]. Over a minimum of 9 months patients are offered at least 13 education and exercise sessions; resulting in at least 16 h of personal support. At the start of the COVID-19 pandemic the DPP transitioned to remote delivery and now includes a self-referral route [6]. It was anticipated that the programme would offer 100,000 places each year and cover the whole of England [7]. A service evaluation of the DPP reported that by December 2018, 324,699 people had been referred. Following referral 47% had attended an initial assessment and 30% had attended at least one group session. Of those who had time to complete the intervention (32,665), 53% completed at least 60% of the sessions and had a mean change in HbA1c of − 2.04 mmol/mol (95% CI; − 1.96, − 2.12). No data were reported on the number of patients who had returned to a HbA1c of below 42 mmol/mol (6.0%) [8].

As previously stated, in Wales there is no equivalent nation-wide programme for people with prediabetes although brief interventions have been available in limited locations, delivered by local General Practice (GP) clusters. A GP cluster is a professional grouping of GP surgeries in a specific geographical location which covers a population of between 30,000 and 50,000, there are 60 GP clusters in Wales. Brief interventions have previously been shown to be beneficial for those with prediabetes [9]. Brunisholz et al. [9] reported on a DPP for patients with prediabetes which included three different pathways. Patients were allowed to enrol in any or all of the pathways, which included a 2-h introductory class, a medical nutrition pathway (MNT) and a weigh to health pathway (W2H). Similar to the NHS DPP both the MNT and the W2H pathways involved multiple, personalised sessions while the 2-h class was a single session taught to a group of patients and included information on healthy eating and physical activity. There were no differences between pathways in achieving the study’s primary outcome, which was a 5% reduction in body mass.

The aim of the current report is to examine the impact of brief lifestyle interventions on HbA1c for people at risk of developing diabetes across two GP clusters in Wales. The NICE guideline PH38 states Brief Interventions can be delivered by general practitioners, healthcare assistants and professionals in primary healthcare and the community, and have the aim of improving diet and increasing physical activity [3]. Success of the interventions will be assessed by change in glycated haemoglobin (HbA1c) and the distribution of patients across the HbA1c normoglycaemic, prediabetes and diabetes ranges 12 months following intervention.

## Method

This report is a pragmatic service evaluation of two brief interventions using routine clinical data. The interventions were independently implemented across two GP clusters, North Ceredigion Cluster network (NCC) and Neath Port Talbot Afan Cluster network (NPT Afan).

### Ethical approval and consent

As a service evaluation, data were limited to secondary use of anonymised data previously collected in the course of normal care. The Health Research Authority’s Decision tool [10] indicated that this evaluation was outside the remit of Research Ethics Committee and full ethical review was not required. As such, this service evaluation was approved by Aberystwyth University Ethical Review Committee as exempt from ethical review and from obtaining informed consent. Data extraction and analysis were in accordance with all relevant guidelines and regulations including the General Data Protection Regulation. All methods, data processing and analysis were in accordance with the Declaration of Helsinki and adhered to Good Clinical Practice guidelines.

### Population

The two clusters serve a combined population of 98,900 (NCC, 48,080; NPT Afan, 50,820). In the NCC cluster 57.8% and in the NPT Afan cluster 26.2% live in a rural area compared to a national average for Wales of 33.9%. The percentage of people living in each cluster classified as being within the most deprived in Wales (within the lowest 20% in Wales) is < 1% in the NCC compared to 47% in the NPT Afan. Of the NCC population 18% are over the age of 65 years and 2.5% over the age of 85 years compared to 18.8 and 2.6% for NPT Afan, respectively (18.7 and 2.5% for Wales) [11, 12]. The NCC cluster contains seven GP surgeries while the NPT contains nine surgeries with each cluster servicing a localised population with the exception of one surgery in the NCC cluster (surgery 16) which is the default surgery for a university student population.

### Intervention

In the NCC patients were offered the brief intervention if they were aged between 18 and 75 years and had a HbA1c between 42 and 47 mmol/mol (6.0–6.4%). In NPT Afan patients were included if they had a previous high blood glucose result, oral glucose tolerance test or HbA1c (i.e. fasting glucose > 6.0 mmol/L; abnormal OGTT; HbA1c > 41 < 48 mmol/mol (> 6.0 < 6.5%)). In both clusters patient data were excluded if they had previously been diagnosed with diabetes.

The interventions in both clusters involved a single face-to-face 15 to 30-min consultation with a health care professional (HCP). The HCP delivering the intervention included health care assistants, practice nurses and general practitioners.

Training needs for HCPs were assessed on an individual basis and when appropriate staff attended the Agored Cymru accredited Level 2 ‘Community Food and Nutrition Skills’ training [13]. This was run by the Community Dietetics Department and is part of the larger ‘Nutrition Skills For Life’ course, which has been developed by public health dietitians in Wales and the Welsh Government to promote evidence based nutrition messages. The training enabled HCPs to develop the competencies required to promote key healthy eating messages.

The style of delivery of the face-to-face consultation was at the discretion of the HCP and was heavily influenced by the patient. During the consultation patients were engaged in conversation about the complications of diabetes, and the benefits of exercise and a healthy diet. Based on the professional judgement of the HCP, patients were signposted to local physical activity groups (e.g. walking clubs), the National Exercise Referral Scheme (NERS) and/or the Foodwise for Life programme. NERS is a structured 16-week supervised exercise programme overseen by a qualified exercise professional [14] and Foodwise for Life is an eight-week evidence-based approach to weight management [15]. On completion of the consultation patients were given information leaflets to reinforce the message on diet and exercise, for the NPT Afan patients this was in the form of the Exeter prediabetes booklet [16]. The primary outcome was glycated haemoglobin (HbA1c) with measures taken at baseline and at 12-month follow-up. In addition to absolute changes in HbA1c at 12-months the proportion of people who had reverted to normoglycaemia (< 42 mmol/mol (6.0%)) or had developed diabetes (> 47 mmol/mol (6.4%)) were also calculated. Data were only included if the patient had attended the brief intervention and both baseline and follow-up data were available.

### Statistics

Data are presented as mean (±SD) unless specified. Differences in HbA1c between primary care clusters were assessed by independent t-tests. Mann-Whitney U tests were also performed to assess robustness of the conclusion to normality of HbA1c distribution. Changes in HbA1c before and after the intervention for the total sample and each surgery were assessed by paired t-tests. Wilcoxon Signed rank tests were also performed to check if the conclusion was affected by the distribution of HbA1c. Twelve months later HbA1c value was recorded and the percentage of patients who reverted to normoglycemia, those who progressed to diabetes, and the associated 95% confidence intervals, were calculated for the total sample, each cluster and each surgery. The statistical significance of changes in glucose tolerance status were assessed by one sample binomial tests through comparison with the hypothesised proportion of people who would i) revert back to normal glucose tolerance naturally without intervention or ii) progress to diabetes, identified through literature review.

To address effects of regression to the mean two approaches were used. The first approach estimated random variation within patients by calculating the within patient variance of the baseline and 12-month HbA1c. This entity was then used to quantify the percentage of observed reduction in 12-month HbA1c due to random variation. Secondly, effects of regression to the mean were assessed by the Roberts (1980) formulae [17] which adjusted baseline HbA1c with the test-retest reliability coefficient and the population HbA1c mean. As the true population HbA1c mean was not known, a range of HbA1c values were tested until changes between 12-month and baseline HbA1c were no longer statistically significant. After adjusting baseline HbA1c, the statistical significance and confidence interval of differences were assessed with paired t-tests.

## Results

The total number of patient data sets analysed was 1207 (592 from NCC and 615 from NPT Afan). At baseline, there was no significant difference in HbA1c between the two clusters (43.87 ± 1.61 mmol/mol (6.16 ± 0.15%) vs 43.83 ± 1.53 mmol/mol (6.16 ± 0.14%), *p* = 0.614) (Table 1). Mann-Whitney U test also showed no significant difference in baseline HbA1c between the two primary care clusters (*p* = 0.82).

**Table 1:** Mean (SD) HbA1c by GP cluster at baseline and follow-up

Cluster (N)		Baseline	Follow-up	Difference	***P*** value (t-test)	***P*** value (Wilcoxon Signed rank test)
		Mean (SD)	Mean (SD)	Mean (95% CI)		
NCC (592)	mmol/mol	43.87 (1.61)	42.59 (4.32)	−1.28 (− 1.61, − 0.95)	< 0.0001	< 0.0001
	%	6.16 (0.15)	6.05 (0.40)	−0.12 (− 0.15, − 0.09)		
NPT Afan (615)	mmol/mol	43.83 (1.53)	40.71 (3.04)	−3.12 (−3.34, −2.89)	< 0.0001	< 0.0001
	%	6.16 (0.14)	5.88 (0.28)	−0.29 (− 0.31, − 0.26)		

*NCC* North Ceredigion Cluster, *NPT Afan* Neath Port Talbot Afan

With data from both clusters combined HbA1c fell from 43.85 ± 1.57 mmol/mol (6.16 ± 0.14%) to 41.63 ± 3.84 mmol/mol (5.96 ± 0.35%), between baseline and follow-up, a decrease of 2.22 mmol/mol (0.20%) (95% CI 2.01 (0.18%), 2.42 (0.22%); *p* < 0.0001). HbA1c in the NCC cluster fell from 43.87 ± 1.61 mmol/mol (6.16 ± 0.15%) to 42.59 ± 4.32 mmol/mol (6.05 ± 0.40%), a decrease of 1.28 mmol/mol (0.12%) (95% CI -1.61 (− 0.15%), − 0.95 (− 0.09%), *p* < 0.0001) (Table 1). HbA1c fell in 408 (69%) patients and in 96 (16%) patients HbA1c increased following the intervention. While in the NPT Afan cluster the HbA1c fell from 43.83 ± 1.53 mmol/mol (6.16 ± 0.14%) to 40.71 ± 3.04 mmol/mol (5.88 ± 0.28%) a decrease of 3.12 mmol/mol (0.29%) (95% CI, − 3.34 (− 0.31%), − 2.89 (− 0.26%); *p* < 0.0001) (Table 1). In 515 (84%) patients HbA1c fell following the intervention and in 53 (9%) patients HbA1c increased. Significant (*p* < 0.001) decreases were observed in 14/16 surgeries according to paired t-tests and 15/16 surgeries according to Wilcoxon signed ranks (Table 2).

**Table 2:** Mean (SD) HbA1c by surgery at baseline and follow-up

Surgery (Cluster)	N	HbA1c (mmol/mol) (%)			
		Baseline	Follow-up	Difference	*P* value (t-test)
		Mean (SD)	Mean (SD)	Mean (95% CI)	
1 (NPT)	88	43.83 (1.61) 6.16 (0.15)	40.67 (2.82) 5.87 (0.26)	−3.16 (− 3.73, − 2.59)	< 0.0001
2 (NPT)	50	44.04 (1.63) 6.18 (0.15)	40.68 (3.30) 5.87 (0.30)	−3.36 (− 4.14, − 2.58)	< 0.0001
3 (NPT)	74	43.86 (1.63) 6.16 (0.15)	41.03 (3.57) 5.90 (0.33)	−2.84 (− 3.59, − 2.09)	< 0.0001
4 (NPT)	18	43.44 (1.38) 6.13 (0.13)	41.28 (2.91) 5.93 (0.27)	−2.17 (−3.35, −0.98)	0.001*
5 (NPT)	124	43.63 (1.36) 6.14 (0.12)	40.57 (2.99) 5.86 (0.27)	−3.06 (− 3.56, − 2.55)	< 0.0001
6 (NPT)	107	43.89 (1.57) 6.17 (0.14)	40.85 (3.14) 5.89 (0.29)	−3.04 (− 3.59, −2.48)	< 0.0001
7 (NPT)	54	43.78 (1.42) 6.16 (0.13)	40.43 (3.13) 5.85 (0.29)	−3.35 (−4.15, − 2.56)	< 0.0001
8 (NPT)	74	43.88 (1.56) 6.17 (0.14)	40.64 (2.51) 5.87 (0.23)	−3.24 (− 3.88, −2.61)	< 0.0001
9 (NPT)	26	44.23 (1.75) 6.20 (0.16)	40.54 (3.04) 5.86 (0.28)	−3.69 (−4.90, − 2.48)	< 0.0001
10 (NCC)	93	44.78 (1.63) 6.16 (0.15)	42.85 (2.64) 6.07 (0.24)	−0.94 (− 1.45, − 0.43)	0.0005**
11 (NCC)	106	43.73 (1.59) 6.15 (0.15)	42.11 (2.27) 6.00 (0.21)	−1.61 (− 2.02, − 1.21)	< 0.0001
12 (NCC)	99	43.72 (1.50) 6.15 (0.14)	42.14 (2.31) 6.01 (0.21)	−1.58 (− 1.97, − 1.19)	< 0.0001
13 (NCC)	54	44.09 (1.56) 6.18 (0.14)	42.15 (2.33) 6.01 (0.21)	−1.94 (−2.61, − 1.28)	< 0.0001
14 (NCC)	115	43.77 (1.60) 6.16 (0.15)	42.37 (2.60) 6.03 (0.24)	−1.39 (− 1.78, − 1.00)	< 0.0001
15 (NCC)	123	44.20 (1.73) 6.19 (0.16)	43.54 (8.14) 6.13 (0.74)	−0.65 (−2.05, 0.75)	0.36**
16 (NCC)	2	44.00 (0) 6.18 (0.00)	44.00 (2.83) 6.18 (0.26)	0.00 (−25.41, 25.41)	1.00

*NCC* North Ceredigion Cluster, *NPT Afan* Neath Port Talbot Afan *P* values from Wilcoxon Signed rank test are the same as t-test except: *0.003; ** < 0.0001;

Of the 592 patients in the NCC, 217 (36.7%) changed category from prediabetes to normal glucose tolerance, 358 (60.5%) remained with prediabetes and 17 (2.9%) developed diabetes, compared to 381 (62.0%), 225 (36.6%) and 9 (1.5%) in NPT Afan, respectively (Table 3). Annual reversion rate to normoglycaemia was estimated to be between 6 and 19%. These estimates were based on both observational studies [18] and data from placebo groups in clinical trials [19, 20], for example the Diabetes Prevention Program Research Group reported 19% of participants in the placebo group who did not have a diagnosis of diabetes but elevated glucose concentrations, reverted back to normoglycaemia [19]. Of the 1207 patients assessed as part of this evaluation, 598 had reverted to normal glucose tolerance at 12 months. The proportion of people who had reverted to normoglycaemia at 12 months (0.50 95% CI 0.47, 0.52) was significantly larger than the lower (0.06 (*p* < 0.0001) and upper (0.19 (*p* < 0.0001)) population estimates. Furthermore, when considered separately the proportion of patients in both NCC (0.37 95% CI 0.33, 0.41) and NPT Afan (0.62 95% CI 0.58, 0.66) were both significantly larger than the lower (0.06; *p* < 0.0001) and higher (0.19; *p* < 0.0001) population estimates (Table 4). Statistical significance of progression to diabetes was assessed through comparison with the rate of natural progression to diabetes, which is reported to range from 5 to 10% [21]. The proportion of people with diabetes at 12 months in the NCC was 0.03 (95% CI 0.02, 0.05) and in NPT Afan 0.02 (95%CI 0.01, 0.03), both significantly smaller than the lower (0.05 (NCC *p* = 0.011; NPT Afan *p* < 0.0001) and upper (0.10 (*p* < 0.0001) population estimates (Table 4).

**Table 3:** Comparison of Glucose Tolerance status at 12 months between two GP clusters

	NCC (***N*** = 592) N (%)	NPT Afan (***N*** = 615) N (%)
NGT (HbA1c < 42 mmol/mol (6%))	217 (36.7)	381 (62.0)
Prediabetes (HbA1c 42–47 mmol/mol (6.0–6.4%))	358 (60.5)	225 (36.6)
Diabetes (HbA1c > 47 mmol/mol (6.4%))	17 (2.9)	9 (1.5)

*NGT* Normal Glucose Tolerance, *NCC* North Ceredigion Cluster, *NPT Afan* Neath Port Talbot Afan

**Table 4:** Proportion of people with normal glucose tolerance and those with diabetes at 12 months

Cluster		NCC	NPT Afan
**Baseline N**		592	615
**Reverted to normoglycaemia**	Proportion (95% CI)	0.37 (0.33, 0.41)	0.62 (0.58, 0.66)
	*P* value hypothesised proportion of 0.06	< 0.0001	< 0.0001
	*P* value hypothesised proportion of 0.19	< 0.0001	< 0.0001
**Developed diabetes**	Proportion (95% CI)	0.03 (0.02, 0.05)	0.02 (0.01, 0.03)
	*P* value hypothesised proportion of 0.05	0.011	< 0.0001
	*P* value hypothesised proportion of 0.10	< 0.0001	< 0.0001

*NCC* North Ceredigion Cluster, *NPT Afan* Neath Port Talbot Afan

The proportion of patients reverting from prediabetes to normoglycaemia at 12 months ranged from 0 to 0.66 between surgeries, while the proportion of patients who developed diabetes ranged from 0 to 0.06 (Table 5). Of the 16 surgeries, 15 had a significantly larger proportion of patients reverting to normoglycaemia than the population estimates of either 0.06 or 0.19 (Table 5). Three of the 16 surgeries had a significantly smaller proportion of patients developing diabetes compared to the population estimate of 0.05 and when compared with the population estimate of 0.10, 13 surgeries had a significantly smaller proportion of people developing diabetes (Table 5).

**Table 5:** Proportion of people with normal glucose tolerance and diabetes at 12 months by GP Surgery

		Reverted to normoglycaemia			Developed diabetes		
Surgery (Cluster)	N	Proportion (95% CI)	*P* value hypothesised proportion of 0.06	*P* value hypothesised proportion of 0.19	Proportion (95% CI)	*P* value hypothesised proportion of 0.05	*P* value hypothesised proportion of 0.10
1 (NPT)	88	0.63 (0.52, 0.73)	< 0.0001	< 0.0001	0 (0, 0.04)	0.03*	0.002*
2 (NPT)	50	0.62 (0.47, 0.75)	< 0.0001	< 0.0001	0 (0, 0.07)	0.10	0.017*
3 (NPT)	74	0.55 (0.43,0.67)	< 0.0001	< 0.0001	0.03 (0.003, 0.09)	0.26	0.029*
4 (NPT)	18	0.50 (0.26, 0.74)	< 0.0001	0.003	0 (0, 0.19)	0.40	0.15
5 (NPT)	124	0.63 (0.54, 0.71)	< 0.0001	< 0.0001	0.02 (0.002, 0.06)	0.06	0.002*
6 (NPT)	107	0.63 (0.53, 0.72)	< 0.0001	< 0.0001	0.03 (0.006, 0.08)	0.21	0.01*
7 (NPT)	54	0.63 (0.49, 0.76)	< 0.0001	< 0.0001	0.02 (0, 0.10)	0.23	0.038*
8 (NPT)	74	0.66 (0.54, 0.77)	< 0.0001	< 0.0001	0 (0, 0.05)	0.04*	0.004*
9 (NPT)	26	0.65 (0.44, 0.83)	< 0.0001	< 0.0001	0.04 (0.001, 0.20)	0.50	0.24
10 (NCC)	93	0.31 (0.22, 0.42)	< 0.0001	0.002	0.03 (0.01, 0.10)	0.29	0.022*
11 (NCC)	106	0.41 (0.31, 0.51)	< 0.0001	< 0.0001	0 (0, 0.03)	0.02*	0.001*
12 (NCC)	99	0.34 (0.25, 0.45)	< 0.0001	0.0001	0.01 (0, 0.06)	0.06	0.002*
13 (NCC)	54	0.35 (0.23, 0.49)	< 0.0001	0.002	0 (0, 0.07)	0.08	0.013*
14 (NCC)	115	0.43 (0.33, 0.52)	< 0.0001	< 0.0001	0.05 (0.02, 0.11)	0.50	0.06*
15 (NCC)	123	0.35 (0.27, 0.44)	< 0.0001	< 0.0001	0.06 (0.02, 0.11)	0.44	0.08*
16 (NCC)	2	0 (0, 0.84)	0.88	0.66	0 (0, 0.84)	0.90	0.81

*NCC* North Ceredigion Cluster, *NPT Afan* Neath Port Talbot Afan. *Significant at 0.05 level

Post hoc assessments of the impact of potential regression to the mean at surgery, cluster and evaluation level estimated the percentage of change from baseline to 12 months due to regression to mean to be 62%. After taking off the effect of regression to the mean, the mean difference between 12 month and baseline HbA1c was − 0.85 mmol/mol (0.08%). Test-retest reliability coefficient between baseline and 12 month HbA1c for the 1207 patients was 0.38. Using Roberts formula [17], provided that the true population HbA1c at baseline was not lower than 40.85 mmol/mol (5.89%), there would be a statistically significant difference (t = − 4.32, df 1206, *p* < 0.0001) of at least − 0.37 (95% CI -0.67, − 0.25) between 12 months and baseline HbA1c overall after adjusting for potential regression to mean.

## Discussion

The effectiveness of 15 to 30-min face-to-face lifestyle interventions for people at risk of diabetes delivered in primary care has been evaluated. These were brief lifestyle interventions and were independently carried out in two primary care clusters. Both intervention programmes were delivered by health care professionals (HCP). In each primary care cluster potential participants with a HbA1c of 42–47 mmol/mol (6.0–6.4%) were identified via database searches, the two cohorts had a similar age profile and were representative of the Welsh population but differed in terms of geography and level of deprivation.

Across both primary care clusters 1207 patients engaged with the interventions and there was a mean decrease in HbA1c of 2.22 mmol/mol (0.20%) 12 months following the intervention. Five hundred ninety-eight patients (0.5) went from prediabetes to normal glucose tolerance, showing that a significantly larger proportion reverted to normoglycaemia as compared with the rate of natural reversion which ranges from 0.06 to 0.19 [18–20]. Results showed significant improvement across the two clusters and also across surgeries in the individual clusters, demonstrating a robust, beneficial outcome in HbA1c from engagement with the intervention in those identified as having prediabetes. Only 26 patients (0.02) developed diabetes in the period for which data were available, a significantly smaller proportion than would be expected, which ranges from 0.05 to 0.10 [21]. Analysis of individual surgeries showed that 13 of the 16 surgeries had a significantly lower number of people developing diabetes when the benchmark value was set at 0.10. If the benchmark was set at 0.05 three of the 16 surgeries had a significantly lower number of people developing diabetes. Taken together these data demonstrate a beneficial outcome for those engaging with this brief intervention which resulted in both a clinically and statistically significant change.

In 2016 the NHS established the Healthier You: NHS Diabetes Prevention Programme (NHS-DPP). Data from the first 2.5 years [8] has shown that of the 32,665 who attended at least one intervention session a mean (95%CI) HbA1c reduction of 1.26 mmol/mol (1.20, 1.31) (0.12% [0.11, 0.12]) was observed and for those who had completed the programme, defined by having attended > 60% of sessions, there was a mean (95% CI) HbA1c reduction of 2.04 mmol/mol (1.96, 2.12) (0.19% [0.18, 0.19]). The single 15 to 30-min brief interventions resulted in comparable changes in HbA1c to the NHS DPP. Compared to the NHS DPP the brief interventions are less resource and time intensive. The key lifestyle messages in both the NHS DPP and the current brief interventions target healthy eating and increasing physical activity. However, the interventions evaluated here link to existing support mechanisms such as local physical activity groups (e.g. walking clubs), the NERS [14] and the Foodwise for Life programme [15], and in doing so offer a less resource intensive option.

This report on two brief interventions has limitations and a deeper and more extensive data extraction alongside the inclusion of a control group would have been preferable. Inconsistencies across GP surgeries in data recording resulted in data on changes in body mass, body composition not being available for inclusion. Uptake rate of the intervention was not recorded and the data presented in the current report includes only patients on whom baseline and follow-up data were available. The data presented may also contain bias as the population on whom data are available may represent those who have a greater motivation for change. Furthermore, the lack of data on referral to existing support services such as NERS and limitations in dietary, physical activity and behavioural change measures do not allow informed conclusions to be drawn on any modifications to lifestyle following the intervention. Despite the limitations, data presented in this report show that the provision of a brief lifestyle intervention to people with prediabetes can result in a significant reduction in HbA1c with a large number of people moving into the normoglycaemic range. This has been evaluated in a large number of people and with different GP surgeries in both a rural location and a population with a high level of deprivation demonstrating that this type of intervention is feasible to deliver, robust and has the potential to be effective in multiple settings and populations. While no cost analysis was included, the scheme is likely to be more cost effective than the NHS DPP.

## Conclusion

The use of a brief intervention delivered in primary care to support people with prediabetes, has the potential to offer a robust and cost-effective alternative to the NHS DPP. These data support the design and implementation of a randomised trial to identify people in which this intervention is most effective, the mechanisms of improvement e.g. increase knowledge, increase self-efficacy or self-management and the resources required to implement on a large scale.

## Data Availability

The datasets used and/or analysed during the current study are available from the corresponding author on reasonable request.

## Abbreviations

DPP: Diabetes Prevention Programme
HbA1c: Glycated Haemoglobin
GP: General Practice
HCP: Health Care Professional
NCC: North Ceredigion Cluster network
NHS: National Health Service
NICE: National Institute for Health and Care Excellence
NPT Afan: Neath Port Talbot Afan Cluster network
PHE: Public Health England
OGTT: Oral Glucose Tolerance Test
QOF: Quality and Outcomes Framework
